# A Few Bad Apples: A Model of Disease Influenced Agent Behaviour in a Heterogeneous Contact Environment

**DOI:** 10.1371/journal.pone.0118127

**Published:** 2015-03-03

**Authors:** Jessica Enright, Rowland R. Kao

**Affiliations:** 1 Boyd Orr Centre, Institute of Biodiversity, Animal Health, and Comparative Medicine, University of Glasgow, Glasgow, United Kingdom; University of Zaragoza, SPAIN

## Abstract

For diseases that infect humans or livestock, transmission dynamics are at least partially dependent on human activity and therefore human behaviour. However, the impact of human behaviour on disease transmission is relatively understudied, especially in the context of heterogeneous contact structures such as described by a social network. Here, we use a strategic game, coupled with a simple disease model, to investigate how strategic agent choices impact the spread of disease over a contact network. Using beliefs that are based on disease status and that build up over time, agents choose actions that stochastically determine disease spread on the network. An agent’s disease status is therefore a function of both his own and his neighbours actions. The effect of disease on agents is modelled by a heterogeneous payoff structure. We find that the combination of network shape and distribution of payoffs has a non-trivial impact on disease prevalence, even if the mean payoff remains the same. An important scenario occurs when a small percentage (called noncooperators) have little incentive to avoid disease. For diseases that are easily acquired when taking a risk, then even when good behavior can lead to disease eradication, a small increase in the percentage of noncooperators (less than 5%) can yield a large (up to 25%) increase in prevalence.

## Introduction

The actions of agents in a disease outbreak are important to the spread and control of that disease. These actions can be viewed as being governed by “games”, i.e. mathematically defined contests amongst individuals, with the aim of understanding how these actions viewed in the context of the population response might influence the spread of that disease. While games played in well-mixed populations are a subject of intense interest [1], considerably less attention has been paid to epidemiological games played in populations with structured contacts.

Strategic games have been of use in studying human disease [2], particularly in modelling vaccination [3] and social distancing during an epidemic [4].

Here, we use a game-theoretic approach to investigate the impact of farmer choice on livestock disease spread in an agricultural setting. Strategic games played between farms present an excellent opportunity for studying games on an explicit contact structure, as interactions typically occur via discretely defined livestock movement patterns [5] or over a limited range of physically possible fenceline contact structures, as farms are embedded in a landscape.

*Game theory* investigates the mathematical dynamics and equilibria of games played by at least two players [6, 7]. In a classical game, players choose actions, and receive payoffs determined by the actions of all players. Game theory is usually used to find the equilibria or show the optimal strategies of a game. Here, using a simulation approach we instead use the concepts to focus on disease outcome, given a method of farmer strategy computation. This simulation approach should find Nash equilibria of the game, but this is mainly of interest in how it contributes to disease prevalence.

Computing the optimal strategies and equilibria of games is often intractable, and is made even more difficult by including many players. *Graphical games* are a class of games in which the payoff for a player is determined by the actions of only a small subset of the players, called that player’s *neighbours*. These relationships can be denoted by a *graph*, or a *network*, consisting of *nodes* joined by *edges*. We define a game played by farms on a graph drawn such that nodes are farms, and two farms are joined by an edge if they share a fence line, and could therefore share disease. For comparison, we also consider a graph in which all farms are joined by edges.

Farmers make many choices that impact their risk for livestock disease. They do not make these choices in isolation, but instead in the context of policy, markets, and the behaviour of their neighbouring farms. These choices can have a significant impact on the spread and prevalence of disease. In this work, we combine a game played on an explicit contact structure with a disease model.

Examples of models incorporating the impact of social structure on disease prevalence are few. Rich *et al.* [8, 9] implement a game theoretic model of farmer behaviour related to foot and mouth disease (FMD) in South America. Farms are arranged in a grid on a torus, with each farmer allowed a choice between a high risk and low risk action in a setting of perfect information. The payoff for a farm is calculated as the mean of the payoffs derived from the game played with each neighbour. Payoffs are structured as for a *stag hunt game*, which has been used in the past to model social cooperations and altruism [10]. Two adjacent farms receive the highest payoff if they both take the low-risk action, and a lesser payoff if they both take the high-risk action. However, if one farmer takes the low-risk action and his neighbour takes the high-risk action, he gets an even lower payoff. This is meant to reflect the higher cost of the low-risk action and the danger of FMD spread from a neighbour.

They find that if there are initial high-risk farms, than all farms will eventually converge to high-risk behaviour, and conclude that it is important to incentivise low-risk behaviours in less-developed areas and industries, as high-risk behaviour areas can serve to spread bad behaviour and, implicitly, disease, to other areas. However, their model does not consider an explicit model of disease spread, which may result in different patterns of behavior, especially if knowledge of the mechanism of disease spread is imperfect, and does not consider the impact of flexible and stochastic payoff structures, two factors we implement here.

Thus our work advances on previous research in several ways in order to simulate a more realistic game: the behaviour of agents is determined by their potentially incorrect beliefs derived from previous turns of the game, the payoffs in the game are determined by the disease state—not directly by the behaviour of other agents, and we consider differing distributions of farmer disease preferences. This combination of evolved and differing preferences and imperfect knowledge is more likely to reflect actual farmer behaviour than previous work on rational agents with perfect information.

## Methods

We implement a simulation of farms in a game theoretic context, with disease spread influenced by farmer choices, and farmer choices influenced by disease spread.

### Game

Here, a *game* is a set of actions, payoffs, and states for some number of players. We consider the disease-risk choices of farmers as actions in a game.

Each of the players in our game is a farm. On every turn each farm is either susceptible or infected. On each turn, a farm chooses to take one of two actions. One action is safer, and one is riskier with respect to the disease. The safer action could involve taking tighter biosecurity measures for example, or buying in certified disease-free animals. Each farm has a payoff for each pair of disease state and action. A payoff might include any of a variety of types of value to a farmer, including financial profit, social value from behaving as peers might prefer, or fulfillment of stewardship responsibilities towards animals.

On each turn, each farm simultaneously makes a choice of action. We simulate the spread of disease given those actions, and then assign payoffs to each farm based on the actions and disease statuses. We assume that the time scales of disease spread and farmer choice are similar; this is consistent, for example, with farmer choices being responsive on the timescale of observed changes in disease status. This choice also shows the effect of farmer behaviour on disease prevalence.

One way in which our game differs from many other games, and in particular the game implemented by Rich *et al.*, is in its stochasticity. We do not deal in expected payoffs directly, but at each turn assign a payoff to a farm depending on its stochastically determined disease status. This noisy signal of infection impacts the development of farmer behaviour. This stochasticity also causes the farmers to have imperfect information: one of our farmers will not know the true expected payoff of an action given a state, only the payoff it receives given that state. Imperfect information and the potential to take a riskier action and not become infected may cause a farmer that will, on average, get a higher payoff from a disease-free status, to develop a preference for riskier actions. This results in substantially different aggregate farmer behaviour than we would see in a deterministic or perfect information system.

### Disease Model

We implement a simple SIS disease model in which every farm is either susceptible to the disease, or infected and infectious.

A farm transitions from susceptible to infected when it is infected by either an infected neighbour or by an outside source associated with the action taken by the farmer (for example, buying-in an infected animal). On a single turn, a farm that has brought-in an infection can then infect a neighbour, but that neighbour cannot then infect a further neighbour that turn.

The probabilities of these infectious events are determined by the individual farm and the actions taken by that farm.

A farm can recover from the infection, becoming susceptible with a probability that is a function of the farm and action taken. Let *f* be a farm, and *G* = (*V, E*) the network of farms, with *f* ∈ *V*. We call the farms that are linked to *f* its *neighbours* and the set of those farms its *neighbourhood*. Let A be the set of all possible actions, D={susceptible,infected} the set of possible disease states.

Our simulation allows farms to have differing probabilities of acquiring the disease in various ways, as well as varying payoffs for the actions and disease states.

At the beginning of each simulation, we assign several parameters to each farm *f* ∈ *V*:

- for each action a∈A and disease state d∈D: a payoff *Y*_(*f, a, d*)_ that *f* would receive if it took action *a* on a turn and ended that turn in disease state *d*,
- for each action a∈A: a probability *P*_(*f, a*)_ that *f* acquires infection from an external source on a turn if it takes action *a*, and
- for each action a∈A: a probability *Q*_(*f, a*)_ that *f* recovers from the disease and becomes susceptible on a turn if it takes action *a*.

In addition, we assign to each edge (*f*_*i*_, *f*_*j*_) ∈ *E*:

- a probability *R*_(*f*_*i*_, *f*_*j*_)_ that, if one of *f*_*i*_ or *f*_*j*_ is infected on a turn, it will infect the other that turn.

We wish to capture the possibility of a continuum of risks, with the majority of them close to the mean, and a few at each extreme—we therefore use truncated Gaussian distributions. Unless stated otherwise, for all farms *f* ∈ *V*, safe action *a*_0_, risky action *a*_1_, and edge (*f*_*i*_, *f*_*j*_) ∈ *E*, we draw the disease spread parameters for each farm from truncated Gaussian between 0 and 1 as in Table 1. In the simulations, we restrict assigned parameter values so that the safe action is always better than the risky action (i.e. for all *f*, only *P*(*f, a*_0_) < *P*(*f, a*_1_) is allowed).

**Table 1:** Default values of *μ* and *σ* used for Gaussian distributions of simulation probabilities throughout this work.

Quantity	*μ* value(s)	*σ*
*P*_(*f, a*_0_)_	0.1	0.1
*P*_(*f, a*_1_)_	0.1	0.95
*Q*_(*f, a*_0_)_	0.2	0.7
*Q*_(*f, a*_1_)_	0.1	0.05
*R*_(*f*_*i*_, *f*_*j*_)_	0.05	0.35

We are interested in regimes of endemic disease where neighbourhood infection can influence a farmers choice between a safe and a risky action. We have therefore chosen to investigate payoffs such that at least some farmer choice will be influenced by neighbours, and avoid payoff structures sufficiently extreme that farmers have a dominating choice regardless of the infections and actions of their neighbours. Similarly, because we are not interested in disease-free or total-disease regimes, we choose parameters to model a moderate prevalence endemic disease, that is, a stable prevalence of greater than 0% and less than 100% percent.

On each turn of the simulation:

- Each farm makes an action choice (the method for this is described in the next Subsection: *Farmer Choice*).
- We simulate farm recovery: for each infected farm *f* that chose action *a*_*i*_ we use *Q*_(*f, a*_*i*_)_ to determine if *f* becomes susceptible.
- We simulate infection from an outside source: for each susceptible farm *f* that chose action *a*_*i*_ we use *P*_(*f, a*_*i*_)_ to determine if *f* is infected from an external source
- We simulate neighbourhood disease spread: for each edge (*f*_*i*_, *f*_*j*_) for which *f*_*i*_ is infected but *f*_*j*_ is not, we use *R*_(*f*_*i*_, *f*_*j*_)_ to determine if *f*_*i*_ transmits the disease to *f*_*j*_
- We give each farm *f* that chose action *a* and is currently in disease state *d* a payoff of *Y*_(*f, a, d*)_.

The payoffs given during the simulation are a function of the farm, action, and disease state. The actions of neighbours only effect a farm’s payoffs in that they might have contributed to the farm’s disease status.

### Farmer choice

Real farmers operate in an environment of uncertainty and constraints. Farmers may form opinions about disease risk based on their personal experience. Farmers can be differently effected by disease. For example, consider bovine viral diarrhoea (BVD), a disease that causes substantial economic loss in Great Britain [12, 13]. The majority of that economic loss is from abortions or other reproductive failures, and therefore BVD is likely to be far more financially damaging to a dairy herd than a beef finishing herd [14]. We have tried to capture some of that uncertainty, imperfect information, and difference in disease effect in our simulated farmers.

Farmer choice is complicated by a number of factors. Our farmers have imperfect information, and their payoffs are only indirectly impacted by their neighbours’ choices, to the extent that these impact their disease status. Initially, they are assumed to be naive, i.e. making choices with little initial information. They gain information in the form of assigned payoffs throughout the simulation.

We implemented two differing ways in which farmers might transform those payoffs into opinions: idealist farmers and realist farmers. Like farmers comparing their situation to government or breed society expectations, our idealist farmers compare their current payoffs to an idealised expected payoff that has been precomputed. In contrast, realist farmers compare the outcome of their current action to the outcomes of their past actions to form an opinion.

Furthermore, we have chosen to have our farmers’ choices only effect their risk for infection from buying-in animals; they are powerless to change their risk for infection from an infected neighbour. This reflects the perceived situation for several major bovine endemics in Great Britain, including bovine tuberculosis [11, 15].

#### Our approach to farmer choice

Every farm maintains three lists of information about its payoffs and preferences. First, each farm starts with an estimate of the expected payoff given its previous disease state, its action choice, and its previous neighbourhood disease state. It does not take into account the possibility of neighbours bringing-in infection and then infecting the farm this turn. For idealist farmers, this expected payoff is the point of comparison to their actual payoff at every stage in the simulation. For realist farmers, this estimate is only used when a farm has no experience of its current situation, and so is generally only used at the beginning of the simulation. Details of these estimates can be found in the attached S1 Appendix: Analytical details of payoff estimates and phase transition points.

Every farm maintains its own estimate of an expected payoff for a given disease state, action choice and neighbourhood state. However, these payoffs are not directly used to choose actions. Instead, we use regret-based ratings derived from those payoffs. Importantly, these ratings are not bounded above or below, which allows a run of good payoffs given an action and state to build up positive feeling about that action. Then a negative outcome for that action may take quite a long time to change the farmer’s belief in the desirability of that action.

We now describe how we calculate the estimated payoffs and action ratings.

Every farm has a record of estimated payoffs given a previous disease status, action choice, and neighbourhood disease status. Idealist farmers do not adjust these over time, instead using the initial expected payoff estimate throughout the simulation. Realist farmers update their estimates by averaging the previous payoff record and the current payoff record for a state—therefore these payoff records are discounted over time. A record for a disease and neighbourhood state is only updated when a farm encounters that state.

Let $Pay_{t}(f,F,a,d)$ be realist farm *f* ∈ *V*’s record of its decayed payoff record for taking action a∈A from disease state *d* with infected members its neighbourhood F prior to time *t*. Consider farm *f* at the beginning of turn *t*: let *d* be its disease state and F be the set of its neighbours that are infected at that time. If it chose to take action *a* on turn *t*, and then ended the turn being given payoff *p*, then before turn *t* + 1, *f* updates:

$$\begin{array}{c} Pay_{t+1}\left(f,F,a,d\right)=\frac{Pay_{t}\left(f,F,a,d\right)+p}{2} \end{array}$$ (1)

The simplicity of the discounting by dividing by two allows us to keep a single number for an estimated payoff for each farm for each action and disease and neighbourhood state. We have left the more memory-intensive computation and storage of a economic-style *β* exponent discounting for future work.

If realist farm *f* did not start turn *t* in disease state *d*, did not take action *a*, or its infected neighbours were not exactly the set F, then it does not change its payoff record for that disease state, action, and infected neighbourhood, and

$$\begin{array}{c} Pay_{t+1}\left(f,F,a,d\right)=Pay_{t}\left(f,F,a,d\right) \end{array}$$ (2)

Finally, every farm maintains a rating for each combination of action and neighbourhood disease status that it encounters in the simulation. Let $Rate_{t}(f,F,a,d)$ be the rating farm *f* holds for action *a* with disease state *d* and infected neighbour set F before turn *t*.

If on turn *t* farm *f* takes action *a* and starts the turn in disease state *d* with exactly F neighbours infected, then:

$$\begin{array}{c} Rate_{t+1}\left(f,F,a,d\right)=\frac{Rate_{t}\left(f,F,a,d\right)}{2}+\left(p_{k}-p_{k}^{'}\right) \end{array}$$

where *p*_*k*_ is the payoff that *f* received on turn *t*, and

$$\begin{array}{c} p_{k}^{'}=\underset{a_{i}\in A}{max}\;\left(Pay_{t}\left(f,F,a_{i},d\right)\right) \end{array}$$ (3)

If *f* does not take action *a*, or did not start turn *t* with farm and disease status *d*, F, then

$$\begin{array}{c} Rate_{t+1}\left(f,F,a,d\right)=Rate_{t}\left(f,F,a,d\right) \end{array}$$

This formulation allows a long run of positive results with a particular action in a set of disease circumstance to build up a high positive rating for that action in those disease circumstances. At the beginning of the simulation all ratings are set to zero.

When making an action choice, a farm checks all actions paired with the current neighbourhood and farm disease status. It then selects the one with the highest rating, breaking ties randomly.

Farms only take into account the disease status of themselves and their neighbours. This serves as an approximation of the sort of local disease prevalence knowledge that a real farmer might have.

### Heterogeneity

Recall that before each simulation we assigned each farm a number of parameters that describe how a farm might acquire, recover from, and pay for disease. We introduce varying levels of heterogeneity into our model in the generation of the payoffs for farms.

We want to simulate two situations: a generally uniform farming industry in which most farms are affected in a similar way by the disease but with some outliers, and an industry that is composed of two different types of farm, one of which is much more affected by the disease than the other. To achieve this, we generate the payoffs drawing from two different distributions:

- a narrow unimodal distribution, here represented as a Gaussian distribution with *σ* of 0.01
- a bimodal distribution, here represented as the sum of two Gaussian distributions with *σ* of 0.01.

We vary the values of *μ* across experiments and for different disease status and action pairs. Unless we state otherwise, we use *μ* values as shown in Table 2.

**Table 2:** Default values of *μ* used for unimodal and bimodal distributions of payoffs when simulating disease spread and behaviour in a generally unified industry as well as an industry composed of equal number of two types of holdings unequally effected by disease.

Action	Disease State	Distribution Type	*μ* value(s)
Safer	Susceptible	Unimodal	0.8
Safer	Infected	Unimodal	0.35
Riskier	Susceptible	Unimodal	0.9
Riskier	Infected	Unimodal	0.45
Safer	Susceptible	Bimodal	0.7 and 0.9
Safer	Infected	Bimodal	0.7 and 0.0
Riskier	Susceptible	Bimodal	0.8 and 1.0
Riskier	Infected	Bimodal	0.8 and 0.1

We also run experiments in which a small percentage of farms have payoffs such that they will always choose the riskier action. This is to simulate a real-life situation in which there is a small group of farmers (“non-cooperators”) who are very resistant to the idea of avoiding the disease. This can be for any of a number of reasons: they may be noncompliant with disease prevention regulations for some reason, they may believe the disease does not effect them, or even that it benefits them to have the disease present. The potential for noncooperators is a concern of any disease control campaign, and so here we evaluate how big a noncooperating group can be before it has a large effect on the overall prevalence of the disease.

### Graphs

We use two different types of graph: a square grid to simulate the geographical layout of farms, and for comparison a complete graph (a clique) in which every pair of farms are neighbours; i.e. a homogeneously mixing farm population with no heterogeneous contact structure. We use graphs of 1024 farms in our simulations, as this is sufficient to detect differences caused by differing parameters, but not so many as to be computationally intractable.

## Results

We report disease prevalences and rates of change in disease status and action choice over simulations with different distributions of payoffs. Reported results are averages of 500 separate simulations.

**Fig 1:**
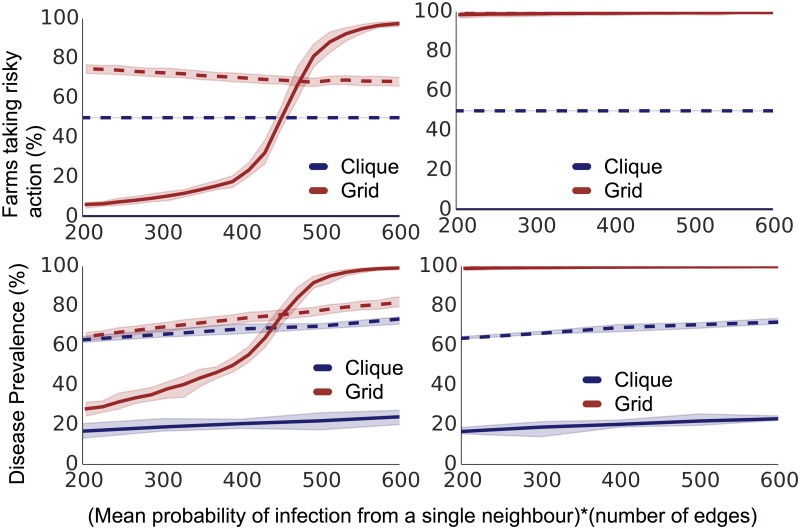
Disease and risky action prevalence after 100 time steps of simulation on a grid graph and a clique graph with bimodal and unimodal distributions of payoffs over a variety of neighbourhood infectiousnesses. In the left column we show results for idealist farms which compare their payoffs to a set general payoff estimate, and on the right realist farms that compare to past payoffs they have experienced. In the top row we show the percentage of farms that are taking the risky action, and in the bottom row the disease prevalence The simulations with unimodal payoff distributions are shown with solid lines, and ones with bimodal distributions in dotted lines. The dotted and solid red lines on the right are identical. Results from simulations on cliques are shown in blue, and results from simulations on grids in red. 95% confidence envelopes are plotted in pale shading around each line.

Fig. 1 shows disease prevalence after 200 time steps of simulation (at which point qualitative patterns of behavior had stabilized) on both the square grid and farm clique with varying probabilities of neighbour infection, considering both unimodal (solid lines) and bimodal (dotted lines) distributions of payoff.

Compared to the other scenarios, which ranked consistently across the infection pressures of interest, simulations of idealist farmers on a grid with a unimodal distributions of payoffs behavior displayed a more marked changes in disease prevalence, with comparatively low prevalence for low neighbour infection probability, but a comparatively high prevalence as the probability of infection increases. The bimodal distribution moderates prevalence on the grid graph.

**Fig 2:**
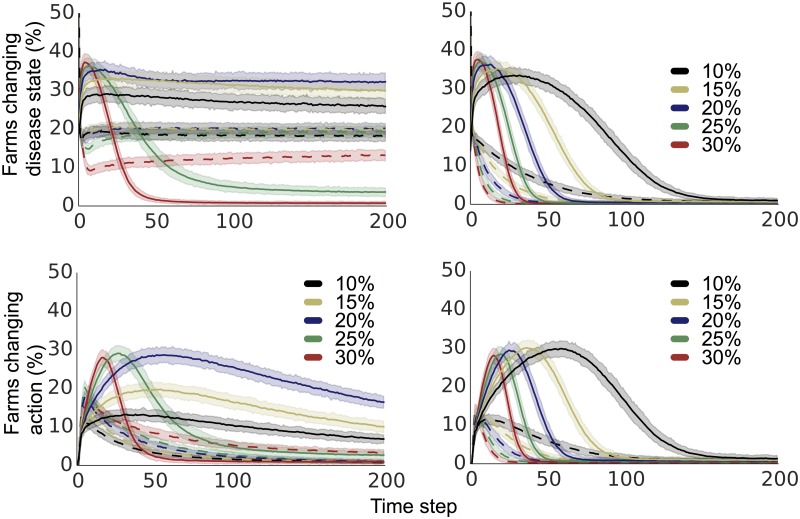
Percentages of farms changing disease and action state over the length of the simulation at differing probabilities of bringing-in the disease with the riskier action, and two distributions of payoffs. In the left column we show results from simulations of idealist farms which compare their payoffs to a set general payoff estimate, and on the right results from simulations of realist farms that compare only to payoffs they have experienced. 95% confidence envelopes are plotted in pale shading around each line.

With more extensive heterogeneity in payoff distributions, some farms are less likely to be swayed by their neighbours actions or disease status. Some farms gain so much by not having disease present that they will almost always take the safer action, even if their neighbours are infected; similarly some farms are unaffected by disease presence, and will never take the safer action. Thus the bimodal distribution moderates prevalence on the grid graph. This effect can be seen in Fig. 2, where we see that increased heterogeneity causes decreased decision-changing. In a heterogeneous situation, farms are more consistent in their decisions, being less responsive to changing disease prevalence in their neighbourhoods. A difference in the distribution of behaviour can drastically change the impact of disease management.

On the other hand, in the simulations run on a clique, the simulations using bimodal distributions of payoffs always have higher prevalence than the simulations using unimodal distributions of payoffs. In the clique simulations with bimodal distribution of payoffs, approximately half of the farms have payoffs such that they make the riskier choice for most of the simulations. The clique structure then means that the remaining farms are more easily infected that they would be in a grid.

We see higher prevalence and risk-taking in a realist rather than an idealist system (Fig. 1): in a realist system farms with a strong negative experience with the safe action early on can be dissuaded from ever taking it again, leading to high prevalence and risk-taking.

### Consistency of Disease and Action

Fig. 2 shows the percentage of farms that change disease status (top) and action choice (bottom) at each time step throughout the simulation for two different distributions of payoffs over several probabilities of bringing-in the disease with the riskier action.

Overall, increased heterogeneity causes a decreased rate of disease status change and a decreased rate of action choice change. This is consistent with farms in a more heterogeneous system being more likely to have extreme payoffs and preferences, and therefore being less likely to be swayed by the disease status of their neighbours. In the homogeneous situation, farms are more easily influenced by infections caught from their neighbours into taking the risky action if the rate of catching the disease from neighbours is high enough, explaining the higher prevalence at higher local infection rates in the homogenous situation.

The exception occurs at high local disease transmission probabilities that result in very high disease prevalence. In the case of very high disease prevalence, it is rare that a farm escapes or recovers from infection. Therefore, few to no farms receive good payoffs for safer behaviour, and all farms quickly switch to choosing the riskier action and continue to do so. Realist farmers change disease state and action less, and have settled to a stable system of all farms infected and taking the risky action before 200 time steps of simulation.

### Small groups of noncooperative farms

Our previous figures have shown populations in which farms drew their payoffs from a symmetric bimodal distribution, composed of the sum of two Gaussians. In effect, this modelled a population of two types of farms, with equal numbers of each type. Here, we present simulations involving a small minority of farms (called *noncooperative farms*) that will always take the riskier action, in order to show the impact on prevalence of a relatively small fraction of noncooperators in a game. How many noncooperators can the game tolerate without a large increase in disease prevalence, and how does that change with different disease transmissibility?

**Fig 3:**
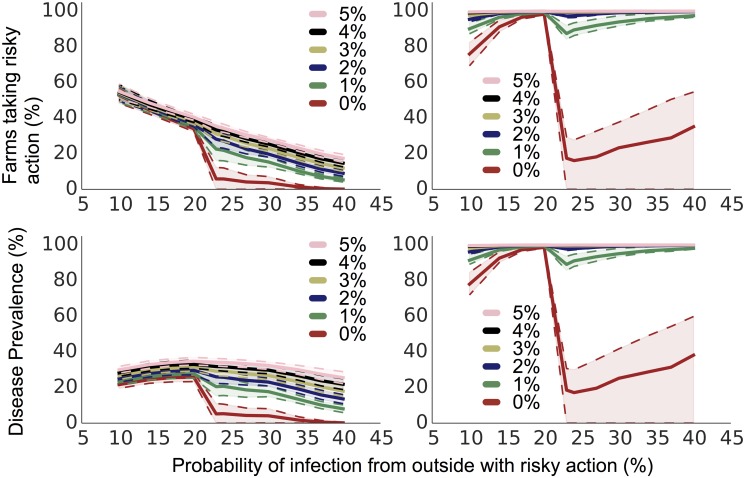
The disease prevalence (*bottom*) and percentage of farms taking the risky action (*top*) over a range of probabilities of bringing in the disease with the risky action. Each line represents a different percentage of noncooperative farms. In the left column we show results from simulations of idealist farms which compare their payoffs to a set general payoff estimate, and on the right results from simulations of realist farms that compare only to payoffs they have experienced. 95% confidence envelopes are plotted in pale shading around each line bounded by dotted lines.

For both realist and idealist settings, and for various proportions of non-cooperators, Fig. 3 shows the proportion of farmers taking the risky action (L) and the disease prevalence (R) as a function of the probability of infection from the risky action (called the *external risk*). Both the disease prevalence and number of farms taking the risky action increase monotonically with the percentage of non-cooperative farmers in both idealist and realist situations. However, disease prevalence has a maximum value for intermediate external risk, with a discontinuity in the behavior of the system. This discontinuity rapidly diminishes with increasing percentages of non-cooperators, effectively disappearing when this reaches only 1 in 20 individuals. In contrast, the percentage of farms choosing the risky action decreases monotonically (Fig. 3), though a similar discontinuity in response is observed.

The external risk at which this discontinuity occurs is determined by the difference in payoffs to the cooperative farmers between the safe and risky actions, i.e. the cost of the safer action (details in S1 Appendix. Analytical details of payoff estimates and phase transition points).

There are effectively two disease regimes, one to either side of the discontinuity. Where external risk is low, the risky action does not have a sufficient penalty in expectation to dissuade most farms. In contrast, where external risk is high, farms may avoid the risky action even if one or two of their neighbours are infected, resulting in lower overall prevalence. Contrast this with the system depicted in Fig. 1, in which higher local disease risk resulted in higher disease prevalence and more risk-taking.

The crucial difference between the two regimes is in the main driver of disease prevalence: at low external risk, disease prevalence is driven mainly by the external risk. At high external risk, disease prevalence is driven mainly by the spread of behaviour and disease from non-cooperators. The non-cooperators are much more important in the high external risk regime. Their influence on the behaviour of other farms can be seen on the left side of Fig. 3, where the different proportions of noncooperators show a much larger impact on overall behaviour in the high external risk regime than in the low external risk regime.

When the external risk is 30%, the difference in disease prevalence between 0% and 5% noncooperators in the idealist simulations is about 23%, with the dramatic (15%) increase when comparing 0% and 1% noncooperators suggesting that the introduction of even a few noncooperators results in a phase transition in prevalence.

The difference between the idealist and realist simulations is striking: we see a much higher overall disease prevalence and a much higher proportion of farms taking the risky action in the realist setting. In the realist setting a small number of early experiences in which a farm becomes infected despite taking the more-expensive safe action can prevent that farm ever taking the safe action again: the payoff estimate for the safe action becomes so low that the farm never takes that action again, therefore never updating its estimate of the payoff for the safe action. Because idealists compare the payoff they have received to an estimated payoff, there remains hope that the safe action is a better option, even if the farm has had a poor payoff taking the safe action in the past.

We investigated the importance of the initial payoff estimates on the overall results. As we would expect, the impact of these changes is much larger in simulations of idealist farmers who use the initial payoff estimates throughout the simulation as a point of comparison to their actual payoffs to generate their ratings for actions. In contrast, the realist farmers quickly discard these initial estimates, and so carrying them has a much smaller impact. In either case, some manipulations of the original payoff estimates have a large impact: if the estimates show the risky action as at least as good as the safe action in the undiseased state, then many farms will take the risky action in their first several actions, giving a very high initial prevalence. This very high prevalence poses a sufficiently high subsequent infection pressure that the safe action is no longer worthwhile for most farms, and the system is overcome with persistent high disease prevalence. We leave a more fine-grained examination of the importance of initial payoff estimates as future work.

We also considered the possibility of geographically clustered noncooperative farms. We found similar results to unclustered noncooperative farmers with a decrease in prevalence with more clustering. The decrease in prevalence is larger in a simulation with a larger number of non-cooperative farms that in a simulation with a smaller number of noncooperative farms. The prevalence decrease is due to the decrease in disease pressure from noncooperative farmers with more clustering: for example, if a noncooperative farm is completely surrounded by other noncooperative farms, that farm does not exert any infectious or behavioural pressure on any other farms.

## Discussion

In contrast to most analyses of human behaviour in epidemiological models, we concentrate on the influence of behaviour trends on disease prevalence, rather than on behaviour equilibria. This would be appropriate, for example, where legislation to control disease is responsive to prevalence, and therefore long term behaviour is a less important measure.

We found that the distribution of payoffs can substantially impact disease prevalences. However, the magnitude and even the direction of that effect depends on the transmissibility of the disease and the arrangement of farmers. Even a small number of noncooperative farmers can make a difference in prevalence, but this contribution is much larger and potentially more important in a disease easily caught from a risky action.

Overall, the distribution of payoffs is important to disease spread. A bimodal distribution of payoffs can result in a much different prevalence to a unimodal distribution of payoffs, even if the mean payoff is the same across the two distributions. An examination of farmer attitudes to disease and the different impact of disease on different farming business models is then an important part of a disease control program. We have used a simple model of farmer belief discounting, leaving the more memory-intensive computation and storage of a economic-style *β* exponent discounting for future work. Similarly, considering systems with different discounting rates for different farmers is left for the future.

Our results provide many interesting insights into the relationships amongst stochastic effects, network relationships and the adoption of risky behaviour. First, where local transmission is clustered (in this case by spatial considerations, but also potentially via social clustering), the variability resulting from local histories of infection can result in a drop in disease prevalence, provided there are sufficient individuals incentivised to take a protective action (and even if some are considerably less inclined). This effect largely disappears where contacts are more homogeneous, which effectively removes the effects of stochasticity (i.e. all individuals see much more similar infection risks).

Second, while there are increased efforts to mobilise social behaviour towards the control disease-related activity (e.g. via adoption of more stringent biosecurity measures, awareness of trade related risks and adoption of voluntary vaccination campaigns), the uptake and therefore impact on disease prevalence can be profoundly influenced by the existence of an external threat, as is for example the case for bovine tuberculosis in Great Britain [15], or BVD in Scotland [16, 17], where in both cases some regions are largely disease-free, but subject to risks of introduction via the importation of livestock from higher incidence areas. These external threats can influence directly farmer behaviour or can themselves be altered via external factors such as legislation.

Intervention efforts should include information for agents about the benefits of the preventative behaviour. When agents only consider their past experiences (as in the case of our realist farmers) and not a true estimate of the benefits of a safe action, disease prevalence increases. This lends importance to efforts by governments and farming organisations to inform farmers about the expected benefits of health schemes, and to provide appropriate incentives to encourage safe behaviour even in the face of poor personal experiences.

Risky behaviour spreads disease, which can, in turn, spread risky behaviour. An understanding of both behaviour and disease parameters is essential to control disease.

## Supporting Information

S1 Appendix Analytical details of payoff estimates and phase transition points. (PDF)
