# Supplementary material for: A Few Bad Apples: A Model of Disease Influenced Agent Behaviour in a Heterogeneous Contact Environment
Source: PLoS One. 2015 Mar 3;10(3):e0118127. doi: 10.1371/journal.pone.0118127 (PMC4348343; doi:10.1371/journal.pone.0118127)
Supplement: S1 Appendix — (PDF) [file pone.0118127.s001.pdf]

Appendix S1. Analytical details of payoff estimates and phase transition points.

Initial estimates of payoffs

Here we describe the initial estimates of payoffs that farms hold. These are only used until the farm has direct experience of an action and are typically only used at the very beginning of a simulation.

Let d_{sus} be the susceptible disease state, and d_{inf} the infected disease state. If farm f is currently susceptible, then the estimated payoff next turn for farm f taking action $a \in \mathcal{A}$ from disease state d_{sus} with currently infected neighbours in set $\mathcal{F}$ is:

$$\left(\prod_{f_h \in \mathcal{F}} (1 - R_{(f,f_h)}))\right)(1 - P_{(f,a)})Y_{(f,a,d_{sus})} \quad + (1 - \left(\prod_{f_h \in \mathcal{F}} (1 - R_{(f,f_h)}))\right)(1 - P_{(f,a)}))Y_{(f,a,d_{inf})} \quad (1)$$

If farm f is currently infected, then the estimated payoff next turn for farm f taking action $a \in \mathcal{A}$ from disease state d_{sus} with currently infected neighbours in set $\mathcal{F}$ is:

$$\left(\prod_{f_h \in \mathcal{F}} (1 - R_{(f,f_h)}))\right)Q_{(f,a)}(1 - P_{(f,a)})Y_{(f,a,d_{sus})} \quad + (1 - \left(\prod_{f_h \in \mathcal{F}} (1 - R_{(f,f_h)}))\right)Q_{(f,a)}(1 - P_{(f,a)}))Y_{(f,a,d_{inf})} \quad (2)$$

Transition to Lower Prevalence

The transition to lower prevalence in Figure 3 happens at about 22% probability of bringing in disease with the risky action over a variety of percentages of non-cooperators, including the situation with no non-cooperators! In fact, it occurs in the same probability of bringing in disease with the risky action in a system of isolated farms with no fence line neighbours. This suggests that the location of this transition is determined only by disease threat from outside, and not from geographic neighbours.

We provide an analytical explanation. Consider a farm with no infection threat from neighbours. Then that farm's choices should take into account only the payoffs and probabilities of bringing in disease with the risky action. At very low additional risk from the risky action, the cost of the action will prevent farmers taking the safe action.

Consider, for example, a disease that would cost a farm £1000. Say the safe action gives 0% chance of bringing in the disease, and the risky action a 1% chance of bringing in the disease, but the safe action costs £500 more than the risky action. It is not worth a farm taking the safe action for only a 1% decrease in the probability of bringing in the disease. However, if the safe action cost only a very small amount, or the risky action increased the probability of bringing in the disease by larger amount, then it would, in expectation, be worth taking the safe action.

The point at which we expect mass switching to the safe action is the point at which the expected payoffs from the safe and risky action are the same to a currently uninfected farm. Then we proceed as below (note that we omit the f in all subscripts for succinctness, and because there is only one farm involved):

$$P_{a_0}Y_{(a_0,inf)} + (1 - P_{a_0})Y_{(a_0,sus)} = P_{a_1}Y_{(a_1,inf)} + (1 - P_{a_1})Y_{(a_1,sus)} \quad (3)$$

$$P_{a_0}Y_{(a_0,inf)} - P_{a_0}Y_{(a_0,sus)} + Y_{(a_0,sus)} = P_{a_1}Y_{(a_1,inf)} - P_{a_1}Y_{(a_1,sus)} + Y_{(a_1,sus)} \quad (4)$$

$$P_{a_0}(Y_{(a_0,inf)} - Y_{(a_0,sus)}) + Y_{(a_0,sus)} = P_{a_1}(Y_{(a_1,inf)} - Y_{(a_1,sus)}) + Y_{(a_1,sus)} \quad (5)$$

In our simulation, we intended the disease to have a cost and taking the safe action to have cost such that these costs combined additively. That is, $(Y_{(a_0,sue)} - Y_{(a_0,inf)}) = (Y_{(a_1,sus)} - Y_{(a_1,inf)})$ and

$(Y_{(a_1,inf)} - Y_{(a_0,inf)}) = (Y_{(a_1,sus)} - Y_{(a_0,sus))}$. We introduce notation for these two costs, saying that:

$$(Y_{(a_0,sus)} - Y_{(a_0,inf))} = (Y_{(a_1,sus)} - Y_{(a_1,inf)}) = \delta_{state} \tag{6}$$

$$(Y_{(a_1,inf)} - Y_{(a_0,inf))} = (Y_{(a_1,sus)} - Y_{(a_0,sus)}) = \delta_{act} \tag{7}$$

Then continuing from Statement 3:

$$-P_{a_0}\delta_{state} + Y_{(a_0,sus)} = -P_{a_1}\delta_{state} + Y_{(a_1,sus)} \tag{8}$$

$$-(P_{a_0}\delta_{state} - P_{a_1}\delta_{state}) = Y_{(a_1,sus)} - Y_{(a_0,sus)} \tag{9}$$

$$P_{a_1}\delta_{state} - P_{a_0}\delta_{state} = \delta_{act} \tag{10}$$

$$P_{a_1} - P_{a_0} = \delta_{act}/\delta_{state} \tag{11}$$

In our non-cooperator simulations, $P_{a_0} = 0, \delta_{act} = 0.1, \delta_{state} = 0.45$ giving a threshold of $P_{a_1} = 0.\overline{22}$. This is consistent with our experimental results.
